# Complete mitochondrial genome of the American bullfrog in Korea, *Lithobates catesbeianus* (Anura: Ranidae)

**DOI:** 10.1080/23802359.2020.1715306

**Published:** 2020-01-20

**Authors:** Jae-I. Moon, Kyo Soung Koo, Hee-Jin Kang, Hye-Rin Park, Ha-Cheol Seong, Dong-Hyun Lee

**Affiliations:** a School of Biological Sciences and Biotechnology Graduate School, Chonnam National University, Gwangju, Korea;; b Research Center of Ecomimetics, Chonnam National University, Gwangju, Korea;; c Department of Biological Sciences, College of Natural Sciences, Chonnam National University, Gwangju, Korea

**Keywords:** *Lithobates catesbeianus* Korea, Ranidae, mitochondrial genome

## Abstract

The complete mitochondrial (mt) genome of *Lithobates catesbeianus* was sequenced and characterized. The circular mt genome was constituted of of 37 genes (13 protein-coding genes, 22 transfer RNAs, and 2 ribosomal RNAs) and a non-coding region (NCR). Phylogenetic analysis based on the full mt genome sequences confirmed that among the genus Lithobates, *L. catesbeianus Korea* is included in a monophyletic group with *L. catesbeianus China,* but not with either *L. catesbeianus Japan* or *L. catesbeianus Canada*. This is the first completed mt genome from *L. catesbeianus Korea*, which provide data for further study of phylogeny in *Lithobates spp.* that have been introduced into a number of different countries originally from North America.

American bullfrogs (Anura) as a member of the family Ranidae resided only in parts of North America, but have been sold around the world for human consumption and are now widely distributed throughout Europe, South America and Asia (Shaw 1802). In Korea, American bullfrogs (*Lithobates catesbeianus*) were first introduced from Japan in 1970 for meat production, but have been recklessly left in the wild due to their lack of economic effectiveness and are known to wreak havoc on ecosystem (Kim et al. 2008; Borzée et al. 2017; Kwon et al. 2017). In 2003, *L. catesbeianus* was designated as invasive species in IUCN Red List of Threatened Species (http://www.iucnredlist.org/). Recently, the research on the impact of American bullfrogs in the ecosystem is drawing keen attention once again since chytrid fungus (*Batrachochytrium dendrobatidis)*, considered as the main culprit behind the rapid decline of the global amphibian species, was reported to have originated in Korea and furthermore, *L. catesbeianus* has been known to play the biggest role in spreading this fungus (Gervasi et al. 2013; Yap et al. 2018; Fu and Waldman 2019). Nearly 50 years after it was introduced to Korea, *L. catesbeianus* has established itself as a native species, but there is still a great lack of genetic research on this species in Korea and its complete mitochondrial (mt) genome has not been reported. Therefore, this study aims to determine the whole mt genome of *L. catesbeianus Korea* and perform phylogenetic analysis with related species, which can help for its phylogenetic position and evolution of genomes.

The *L. catesbeianus* specimen was collected from the southern coast of Korea (36.59 N, 127.30E). We extracted the genomic DNA from the subsample (muscle) using the DNeasy Blood & Tissue kit (Qiagen, Valencia, CA) according to the manufacturer’s protocol, and the extracted DNA sample was deposited at the Museum of Wildlife, located in Research Center of Ecomimetics, Chonnam National University, Korea (Specimen accession number: 2019-RCE-LC017). We determined the complete mt genome sequence using the next-generation sequencing reads (400-bp length in each read) generated from MiSeq (Macrogen, Seoul, Korea). Mapped reads were used for *de novo* assembly and annotation by using commercial software (MITOS) to identify the full mt genome with about an average 150 x coverage.

The complete mt genome of *L. catesbeianus* was 17,603 bp in length deposited in GenBank (Accession No. MN241124), and contains 13 protein-coding genes (PCGs), 22 transfer RNA (tRNA) genes, 2 ribosomal RNA genes (srRNA and lrRNA), and a putative long non-coding control region (NCR). 12 protein-coding genes, 14 tRNAs, and 2 rRNAs were predicted to be transcribed from same strand (heavy strand), whereas 1 protein-coding gene (NADH dehydrogenase subunit 6) and 8 tRNA genes (*tRNA^Gln^*, *tRNA^Ala^*, *tRNA^Asn^*, *tRNA^Cys^*, *tRNA^Tyr^*, *tRNA^Ser^*, *tRNA^Pro^*, and *tRNA^Glu^*) were encoded on the light strand. The nucleotide composition of the *L. catesbeianus Korea* (A = 28.6%, C = 25.9%, G = 14.2%, and T = 31.2%) was similar to that of *L. catesbeianus China* (A = 28.6%, C = 26.0%, G = 14.1%, and T = 31.3%) and *L. catesbeianus Japan* (A = 28.7%, C = 25.8%, G = 14.2%, and T = 31.3%). The sequence comparisons between *L. catesbeianus Korea* and *L. catesbeianus China* indicated a 99.81% sequence identity, but sequence identity between *L. catesbeianus Korea* and *L. catesbeianus Japan* was 99.54%, which place the *L. catesbeianus Japan* sister to these two species (Figure 1).

**Figure 1.**
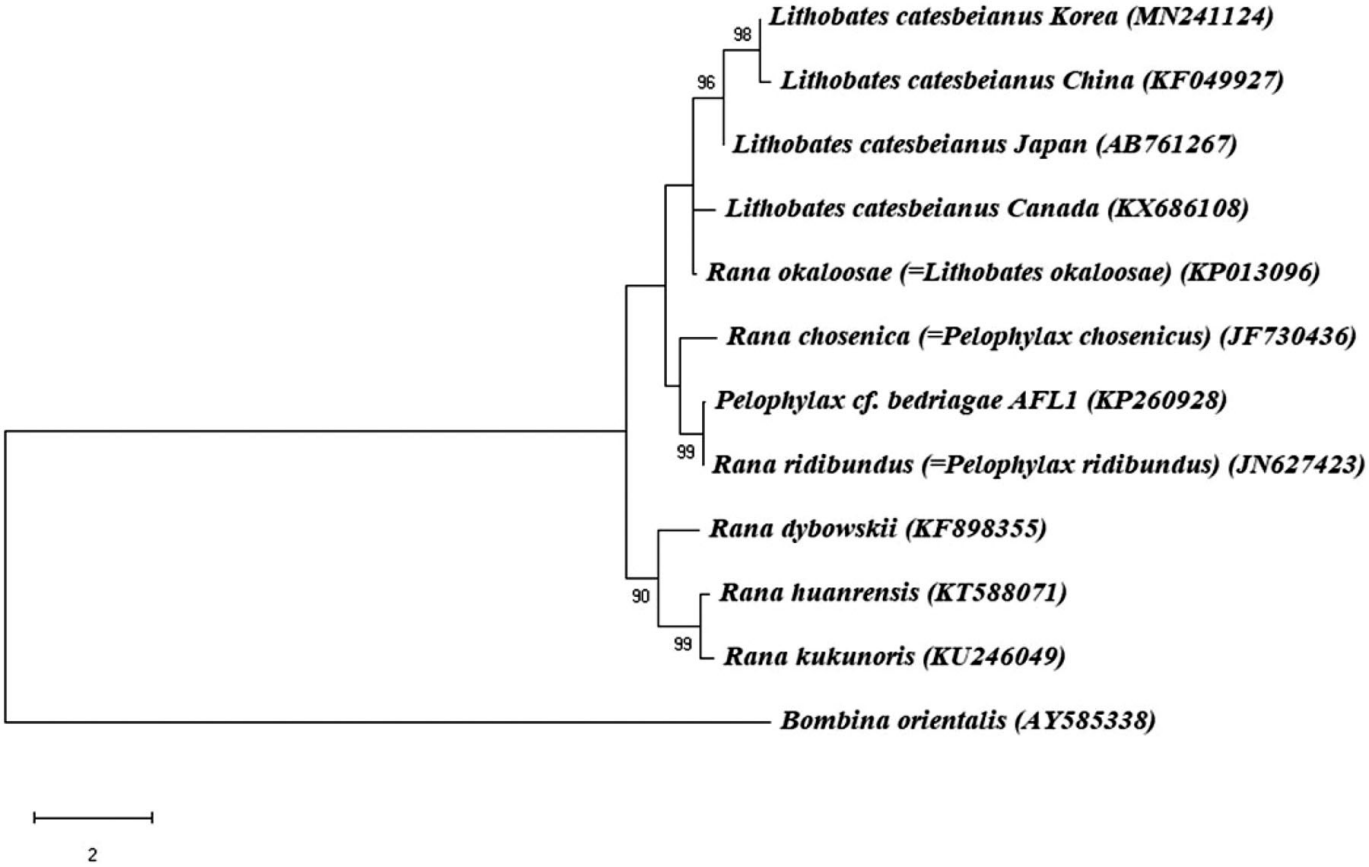
Phylogenetic tree of *Litobates catesbeianus* and other related species based on complete mitochondrial (mt) genome data. The bootstrap value based on 1000 replicates is shown on each node. *Bombina orientalis* was used as outgroup for tree rooting. The phylogenetic analysis was performed using MEGA7 (Saitou and Nei 1987).

In order to investigate the phylogenetic position of *L. catesbeianus Korea*, the full mt genome sequences of twelve Amphibia species were extracted from Genbank, and *Bombina orientalis* served as outgroup. It is intriguing to note that *L. catesbeianus Canada* does not share close relationship with other three *L. catesbeianus* species in Asia, which is probably due to a long period of geographical isolation (Figure 1). Furthermore, *L. catesbeianus Korea,* originally stemmed from Japan, shares closer relationship with *L. catesbeianus China* in a monophyletic group than with *L. catesbeianus Japan* (Figure 1). These data provide important molecular data for further evolutionary analysis for the phylogenetic relationships of the family and also useful genetic marker for identification and ecological studies on *L. catesbeianus,* native to many countries.

## Data Availability

GenBank accession number from the complete mitochondrial genome of *Lithobates catesbeianus* (MN241124) has been registered with the NCBI database.
